# Gut microbiota promoting propionic acid production accompanies caloric restriction-induced intentional weight loss in cats

**DOI:** 10.1038/s41598-024-62243-4

**Published:** 2024-05-24

**Authors:** J. C. Rowe, J. A. Winston, V. J. Parker, K. E. McCool, J. S. Suchodolski, R. Lopes, J. M. Steiner, C. Gilor, A. J. Rudinsky

**Affiliations:** 1 Department of Veterinary Clinical Sciences, The Ohio State University College of Veterinary Medicine, Columbus, OH USA; 2 Comparative Hepatobiliary Intestinal Research Program (CHIRP), The Ohio State University College of Veterinary Medicine, Columbus, OH USA; 3 Department of Clinical Sciences, North Carolina State University College of Veterinary Medicine, Raleigh, NC USA; 4 Gastrointestinal Laboratory, Department of Small Animal Clinical Sciences, Texas A&M University College of Veterinary Medicine, College Station, TX, USA; 5 Department of Small Animal Clinical Sciences, University of Florida College of Veterinary Medicine, Gainesville, FL USA

**Keywords:** Obesity, Microbiome, Microbial ecology, Obesity

## Abstract

Rodent models and human clinical studies have shown gut microbiota-derived short-chain fatty acids (SCFAs) play roles in obesity and insulin resistance. These roles have been minimally explored in cats, where in the USA an estimated 60% of cats are overweight or obese. Overweight/obese research cats (n = 7) were transitioned from a maintenance diet to a reduced calorie diet fed ad libitum for 7 days, then calories were restricted to achieve 1–2% weight loss per week for an additional 77 days. Cats then received their original maintenance diet again for 14 days. Significant intentional weight loss was noted after calorie restriction (adjusted p < 0.0001). 16S rRNA gene amplicon sequencing and targeted SCFA metabolomics were performed on fecal samples. Fecal microbial community structure significantly differed between the four study phases (PERMANOVA p = 0.011). Fecal propionic acid was significantly higher during caloric restriction-induced weight loss (adjusted p < 0.05). Repeated measures correlation revealed the relative abundances of *Prevotella 9 copri* (correlation coefficient = 0.532, 95% CI (0.275, 0.717), p = 0.0002) significantly correlated with propionic acid composition. Like humans, obese cats experienced an altered microbial community structure and function, favoring propionic acid production, during caloric restriction-induced weight loss.

## Introduction

The gut microbiota comprises a dynamic community of microbes within the intestinal tract that influences their host in states of health and disease through production of microbial-derived metabolites. In people, deviations from the normal microbiota and alteration of microbial-derived metabolites, including short-chain fatty acids (SCFAs), are well-demonstrated in the context of obesity and are influenced by diet^1^. Recent research aims to pinpoint specific drivers of these interactions with meta-analysis revealing significantly greater amounts of the SCFAs acetate, butyrate, and propionate in feces from obese people compared to nonobese controls^2^. Functionally, SCFAs play diverse roles in the host and can both serve as a source of energy for host cells, promoting adiposity, as well as stimulate pancreatic β-cell insulin secretion, appetite regulating hormone induction, and glucagon-like peptide 1 (GLP-1) activity via receptor-mediated mechanisms^3–5^. Disentangling these complex interactions is a frontier for obesity therapeutic interventions along with obesity-related disease states including insulin resistance in type 2 diabetes^6^.

Obesity is not an epidemic limited to people, with the prevalence of overweight or obese cats reaching 63% in one study^7^ and 41% in a population of over 9062 cats presented to a veterinary referral hospital^8^. Previous work has demonstrated that diet and weight loss improve insulin sensitivity of obese cats^9^. Though less extensively studied, the gut microbiota of cats is also known to be sensitive to diet composition^10^. Few investigations have targeted dietary influence on microbial SCFA production in cats, with limited data suggesting multiple forms of fiber supplementation yield increased SCFAs recovered in feces^11–15^. This is unsurprising given that SCFAs arise from breakdown of nondigestible polysaccharides from supplemented fermentable fiber^6^. Importantly, most feline disease states, including obesity, have not been characterized in the context of gut microbiota mediated SCFA production, with the exception of chronic kidney disease (CKD) where fecal isovaleric acid was shown to be increased in cats with CKD^16^. Characterization of diet, gut microbiota, and SCFA interactions in obese cats who successfully lose weight may both provide insight to better combat obesity in cats and identify broader metabolic links that apply translationally across medical science.

With the increasingly recognized role of the gut microbiota and microbial-derived SCFAs regulating host metabolism in the context of obesity, the aim of this study was to characterize changes in the gut microbial composition and fecal SCFAs during intentional, caloric restriction-induced weight loss of obese and overweight cats. We hypothesized that the gut microbial composition would be altered during a period of caloric restriction-induced, intentional weight loss and accompanied by an altered fecal SCFA profile driven primarily by the dietary change as well as the change in host obesity status that occurs from caloric restriction. Further, we hypothesized that when a weight loss phase provision of restricted dietary energy is discontinued and a maintenance level of dietary energy provided with the original maintenance diet is reintroduced, observed changes to the gut microbiota and fecal SCFA profile during weight loss will not be sustained primarily as a result of the diet change.

## Materials and methods

### Animals, housing, dietary composition, and fecal collection

The study protocol was approved by The Ohio State University Institutional Animal Care and Use Committee (2014A00000076), and all experiments were performed in accordance with relevant guidelines and regulations. The study findings are reported adhering to ARRIVE guidelines^17^. Seven purpose-bred cats owned by The Ohio State University were used in this study, including five castrated males and two spayed females. Prior to introduction to the animal facility, all cats received prophylactic treatment for endoparasitism. Routine fecal examination for the presence of gastrointestinal parasites was performed every 6 months while cats remained in the animal facility. At the start of the study all cats were 4 years old and body condition score (BCS) was assessed to be overweight (BCS 6 or 7/9) or obese (BCS 8 or 9/9)^18,19^. Specifically, BCS was 7/9 in three cats, 8/9 in three cats, and 9/9 in one cat. Cats were group-housed in facilities accredited by the Association for Assessment and Accreditation of Laboratory Animal Care. Cats were housed in two separate rooms (4 cats in one room and 3 cats in the other) and randomly divided into 2 treatment groups (groups A and B). All investigators were aware of the housing groups throughout the study and were not blinded to this allocation. The study was started with two cats per room (group A) while the remaining cats (group B) were maintained on their maintenance diet fed ad libitum. Once group A completed the full study period, group B then began their study period. This approach was selected to minimize room and/or time effects that could be introduced. Cats included in this study were specifically acquired as an overweight/obese colony and thus respresent a model of chronic obesity. Cats were acclimatized and socialized for more than a year before the start of experiments with environmental enrichment provided. All cats remained overweight or obese during this extended acclimatization period. Examination by veterinarians along with routine laboratory tests, including complete blood counts, serum chemistry profiles, and urinalysis were performed at the beginning of the experiment, and all cats were considered healthy except for being overweight or obese.

**Figure 1:**
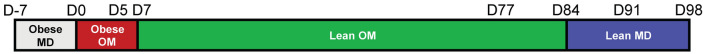
Schematic of study phase timeline. Study days of fecal sample collection are listed across the top of the figure. The colored bars represent the four study phases: Obese MD (gray), Obese OM (red), Lean OM (green), and Lean MD (blue), and are proportional in length to the study timeline.

This study was a repeated measure cross-over design. Each cat underwent four consecutive phases of dietary treatments in the following order: (1) Maintenance diet (Purina^®^ Friskies^®^ Classic Paté Mariner’s Catch^®^, Nestlé Purina PetCare, St. Louis, MO) fed ad libitum for 2 weeks (Obese MD), (2) Veterinary therapeutic weight loss diet (Purina Veterinary Diets^®^ OM Overweight Management^®^ Feline Formula, Nestlé Purina PetCare, St. Louis, MO) fed ad libitum for 1 week (Obese OM), (3) Veterinary therapeutic weight loss diet (OM Overweight Management^®^) with caloric restriction to achieve 1–2% reduction in body weight per week (Lean OM) for 11 weeks, (4) Maintenance diet (Friskies^®^ Classic Paté Mariner’s Catch^®^) fed for 2 weeks (Lean MD). Body condition was monitored by measurement of body weight and BCS. The study design is illustrated in Fig. 1. Macronutrient compositions of study diets are shown in Supplementary Table S1 and average caloric dietary intake through the four experimental phases are reported in Supplementary Table S2 for each individual cat.

Cats were fed twice daily in separate cages and were allowed an allotted time to finish meals (2 h for the morning meal, 14 h for the evening meal). A weighed portion of food was offered, and at the end of the feeding period the residual amount was recorded. For the first two study phases (Obese MD and Obese OM), the diet was offered ad libitum. The diet was then restricted during the third study phase (Lean OM). During the calorie restricted feeding period of controlled weight loss, diet consumption was converted to a caloric intake estimation, then was adjusted weekly by a board certified veterinary nutritionist (V. Parker) based on a previously described dietary management strategy to achieve weight loss with a rate of approximately 1–2% of body weight per week^18,19^. During the final study phase (Lean MD), the original maintenance diet was reintroduced and fed at a caloric amount to maintain body weight. Body weight was measured weekly.

Standardized meal tests were conducted following withholding food for a 12 h period at the conclusion of each study phase as previously described for a separate study in order to evaluate incretin hormone responses^20^. Briefly, 40 g of a palatable canned diet (Hill’s Prescription Diet a/d Canine/Feline Canned, Hill’s Pet Nutrition, Topeka, KS) was offered for a duration of 10 min. Following the 10 min period, any remaining diet was removed, weighed, and recorded.

Fresh, naturally-voided fecal samples were collected and then flash-frozen upon collection and stored at − 80 °C. Samples were collected on days − 7, 0, 5, 7, 77, 84, 91, and 98 (Fig. 1). Fecal samples were collected within three hours of natural voidance, with overnight study personnel present in the animal facility to ensure timely collection. Fecal samples were manually homogenized with aseptic technique prior to flash freezing. Infrequently, a fresh, naturally-voided fecal sample could not be obtained from a cat on all sampling days, including day − 7 for cats 4 and 6, day 5 for cat 1, day 91 for cat 1, and day 98 for cat 6. In downstream analyses, these individual time points were treated as missing samples, thus no values for either microbiome or SCFA data are reported or analyzed for these cats at those timepoints. All samples were batch analyzed at the end of the collection period.

All cats were adopted out to forever homes following completion of the study to familes where they would live the remainder of their lives as a household pet.

### DNA extraction and 16S rRNA gene amplicon sequencing

DNA extraction and quantification was performed for all fecal samples through the University of Michigan Microbiome Core. DNA was extracted via a MagAttract PowerMicrobiome kit (Qiagen, Germantown, MD, USA) per manufacturer instructions. Aliquots of each sample during the DNA extraction process were performed using an Eppendorf EpMotion liquid handling system (Eppendorf, Enfield, CT, USA). Once extraction was complete, 1 μL aliquots from each extraction were quantified using a Quant-iT PicoGreen dsDNA Assay fluorometric kit (ThermoFisher Scientific, Waltham, MA, USA) prior to 16S rRNA gene amplification. The V4 region of the 16S rRNA gene was then amplified with primers 515f and 806r^21^ utilizing a dual indexing approach^22^. The components of the PCR master mix used were 2 μL 10× AccuPrime PCR Buffer II (ThermoFisher Scientific), 11.85 μL double-distilled water, 0.15 μL AccuPrime High Fidelity Taq Polymerase (ThermoFisher Scientific), 1 μL of each extracted DNA sample, and 5 μL of a 4 μM solution of each primer. For PCR amplification, an initial 120 s cycle at 95 °C was followed by 30 amplification cycles with the following settings: denaturation at 95 °C for 20 s, annealing at 55 °C for 15 s, and then 72 °C for 900 s. Following amplification, PCR products were held at 4 °C until further analysis. All PCR products were visualized using an E-Gel 96 with 2% SYBR Safe DNA Gel Stain (ThermoFisher Scientific). Negative quality controls through this process included sterile extraction reagents and master mix^23^.

Normalization of amplicon libraries utilized a SequalPrep Normalization Plate Kit (Life Technologies, Carlsbad, CA, USA) per manufacturer protocols for sequential elution. The final library size consisted of equimolar amounts of each sample normalized to the lowest sample concentration following using a Kapa Biosystems Library Quantification kit for Illumina platforms (Kapa Biosystems, Wilmington, MA, USA). Amplicon sizes within the pooled library were determined with an Agilent Bioanalyzer High Sensitivity DNA analysis kit (Agilent Technologies, Santa Clara, CA, USA). An Illumina MiSeq platform performed paired-end, de novo amplicon sequencing using a MiSeq reagent kit (Illumina, San Diego, CA, USA) with V2 chemistry for 500 cycles for 2 nM and 4 nM libraries per manufacturer instructions with previously described modifications^22^. Library diversity was created with a 15% PhiX spike (Illumina) to yield a final library with load concentration 5.5 nM. Each reagent used for Illumina sequencing was prepared per Illumina manufacturer recommendations for the MiSeq personal sequencer system^22^. Before sequencing, the Illumina reagent cartridge was loaded with custom read 1, custom read 2, and index primers. Sterile extraction reagents and master mix were negative quality controls for sample sequencing^23^.

### Fecal microbiota analysis

Sequence analysis of the V4 region of the 16S rRNA gene amplicons was performed through R Studio (Version 2022.07.1, Build 554)^24,25^. Contigs were assembled from the paired-end reads and subsequently trimmed, filtered, and converted into amplicon sequence variants (ASVs) through the DADA2 pipeline (Version 1.26.0)^26^. Before taxonomy was assigned, chimera sequences were identified and removed as well as any ASVs with lengths < 250 base pairs or > 256 base pairs. Following this, ASVs were assigned taxonomy using the SILVA 16S rRNA Sequence Data Base (Version 138.1)^27^. Taxonomy table generation was performed using the phyloseq package (Version 1.42.0) in R Studio^28^.

Evaluation of alpha and beta diversity was performed in R Studio with phyloseq (Version 1.42.0) and vegan (Version 2.6-4) packages^28,29^. Alpha diversity metrics for each sample in each of the four study phases were calculated with three methods: Shannon Index, Inverse Simpson, and observed ASVs. The Shannon Index was then transformed into EXP (Shannon) (e^H). The alpha diversity metrics by study phase were then visualized using GraphPad Prism (Prism 10 Version 10.0.0 for macOS, GraphPad Software LLC, La Jolla, CA, USA). Beta diversity was assessed via Bray–Curtis dissimilarity distance matrix generated and visualized with non-metric multidimensional scaling (NMDS) approach in R Studio with stress values of < 0.2 considered acceptable^30^. An outlier (day 5 sample from cat 7) was found to separate from all other samples and was noted to have 4013 reads when all other fecal samples had at least 15,000 reads, so this single data point was removed from all analyses.

Relative abundances at both the phylum and family level were visualized with GraphPad Prism. All phyla and families reaching an abundance of at least 1% within a sample were represented, whereas any phyla or families of ASVs not reaching 1% abundance within a sample were placed in a separate category of ASVs less than 1% abundant. The mean relative abundance by study phase at the family level was visualized using ggplot2 (Version 3.4.0) in R Studio.

### Fecal short-chain fatty acid measurement

Targeted metabolomics were utilized to assess the fecal SCFAs propionic acid, acetic acid, butyric acid, valeric acid, isobutyric acid, and isovaleric acid. A modified version of previously described protocol^31^ was utilized as follows: 0.5 g of feces were mixed with 4.25 mL of deionized water and 250 μL of a premade solution of an internal standard of 140 mM 4-methylvaleric acid in formic acid (Helsinki, Finland). The mixture was then vortexed for 4 min and centrifuged at 5000×*g* at 4 °C for 15 min, followed by additional centrifugation of 1 mL of the supernatant at 10,000×*g* at 4 °C for 10 min^31,32^. The clear supernatant was then filtered into a 1.5 mL crimp vial by using a syringe filter (Acrodisc LC 13 mm with a 0.2-μm polyvinylidene fluoride membrane, 4450 T, Pall Corporation, Port Washington, NY, USA). For measuring concentrations, 1 μL of the filtrate was used for gas chromatography with a flame ionization detector (Agilent 7890A and 7683, Agilent Technologies, Espoo, Finland). Individual SCFA concentrations are reported as μmol/mL of fecal content.

### Statistical analysis

Change in body weight over the duration of the study was characterized as percentage of the original body weight for each individual cat. Within GraphPad Prism, percentage change in body weight was assessed to be normally distributed via Shapiro–Wilk test and multiple comparisons were then conducted via one-way analysis of variance (ANOVA) with a false discovery rate (FDR) post-hoc test. All FDR post-hoc tests performed throughout this study used the Benjamini, Krieger, and Yekutieli method^33^, and all normality testing used the Shapiro–Wilk method. Alpha diversity metrics were assessed for normality and determined not to be normally distributed. The non-parametric Kruskal–Wallis test was then applied with a FDR post-hoc test. To assess differences in beta diversity between the four study phases permutational multivariate ANOVA (PERMANOVA) was utilized through the phyloseq and vegan packages in R Studio. The same analysis was performed to ensure there were no statistically significant differences in beta diversity between individual cats (PERMANOVA p = 0.549).

Differentially abundant taxa were identified with Linear discriminant analysis Effect Size (LEfSe, Galaxy Version 1.0)^34^. A threshold LDA score of 2.0 was used in the “one-against-all” test parameter, which was selected to assess for taxa that would be distinctive in any one of the four study phases, and the alpha value for Kruskal–Wallis test at 0.05. ASVs identified as differentially abundant via LEfSe were not normally distributed. Thus, the relative abundances of differentially abundant ASVs were further assessed in each study phase through multiple comparisons testing performed with Kruskal–Wallis test and FDR post-hoc test in GraphPad Prism. Differentially abundant taxa were also identified by using the DESeq2 package (Version 1.38.0) in R Studio^35^. Parameters were set to Test = Wald, FitType = Parametric, Cook’s Cutoff = FALSE, independentFiltering = FALSE (i.e., not applied to the dataset), and Benjamini–Hochberg post-hoc correction was used to generate false discovery rate adjusted p values similar to previously described^23^. Comparisons were made on a log_2_ fold change basis from the Obese MD study phase to the other three study phases (Obese OM, Lean OM, and Lean MD) to identify either significantly decreased or enriched abundance of individual ASVs. These results were visualized using the package EnhancedVolcano (Version 1.16.0).

SCFA concentrations were not normally distributed; thus SCFA concentrations during each study phase were assessed using Kruskal–Wallis test and FDR post-hoc test in GraphPad Prism. The percentage composition of individual SCFAs for each fecal sample were also calculated by dividing the concentration of an individual SCFA by the sum of all SCFA concentrations in the same sample as previously described^36–38^. These values were normally distributed and a one-way ANOVA test with FDR post-hoc test applied. Further, the mean percentage composition for each SCFA during each study phase was also calculated.

MetaboAnalyst (Version 5.0) was used to further analyze concentrations of measured SCFAs in fecal samples. Concentrations were utilized and not subjected to additional filtering or normalization. Principal component analysis was then performed, which utilized the prcomp package as well as the R script chemometrics.R. A Random Forest machine learning algorithm was then applied to the same data using the randomForest package. Significant features in the Random Forest analysis were determined using the mean difference in classification accuracy when permuted. Versions of prcomp, chemometrics.R, and randomForest were contained within MetaboAnalyst (Version 5.0). All MetaboAnalyst analysis steps used R Version 4.2.2.

Repeated measures correlation using the rmcorr package (Version 0.6.0)^39^ was performed in R Studio utilizing code obtained and modified from the web-based platform rmcorrShiny^40^. Data from all phases of analysis that contained paired relative abundance and SCFA measurements were utilized, including measurements of zero relative abundance. The correlation analysis was then visualized using ggplot2 in R Studio. Prior to correlation analysis, mathematical outlier testing to identify extreme relative abundance percentages was determined using GraphPad Prism with the ROUT method and Q = 1%. For ASV 71, the relative abundance values of 49.8%, 22.8%, 19.3%, and 6.3% were mathematical outliers. For ASV 372, the relative abundance values of 0.76%, 5.8%, and 16.5% were mathematical outiers. Though mathematically outliers by percentage relative abundance, these values were retained in the correlation analysis given the compositional nature of amplicon data, as percentage composition cannot be directly translated to absolute microbe abundance^41^.

Missing data points, either as a result of lack of fecal sample from a cat on a specific day or from the outlier 16S amplicon sample that was removed due to low read count, were treated as true missing samples and were not treated as having a value of zero. Therefore, in areas of multiple comparisons between study phases there are either 12 or 13 values for individual groups.

## Results

### Caloric restriction-induced weight loss occurs following calorie restriction

**Figure 2:**
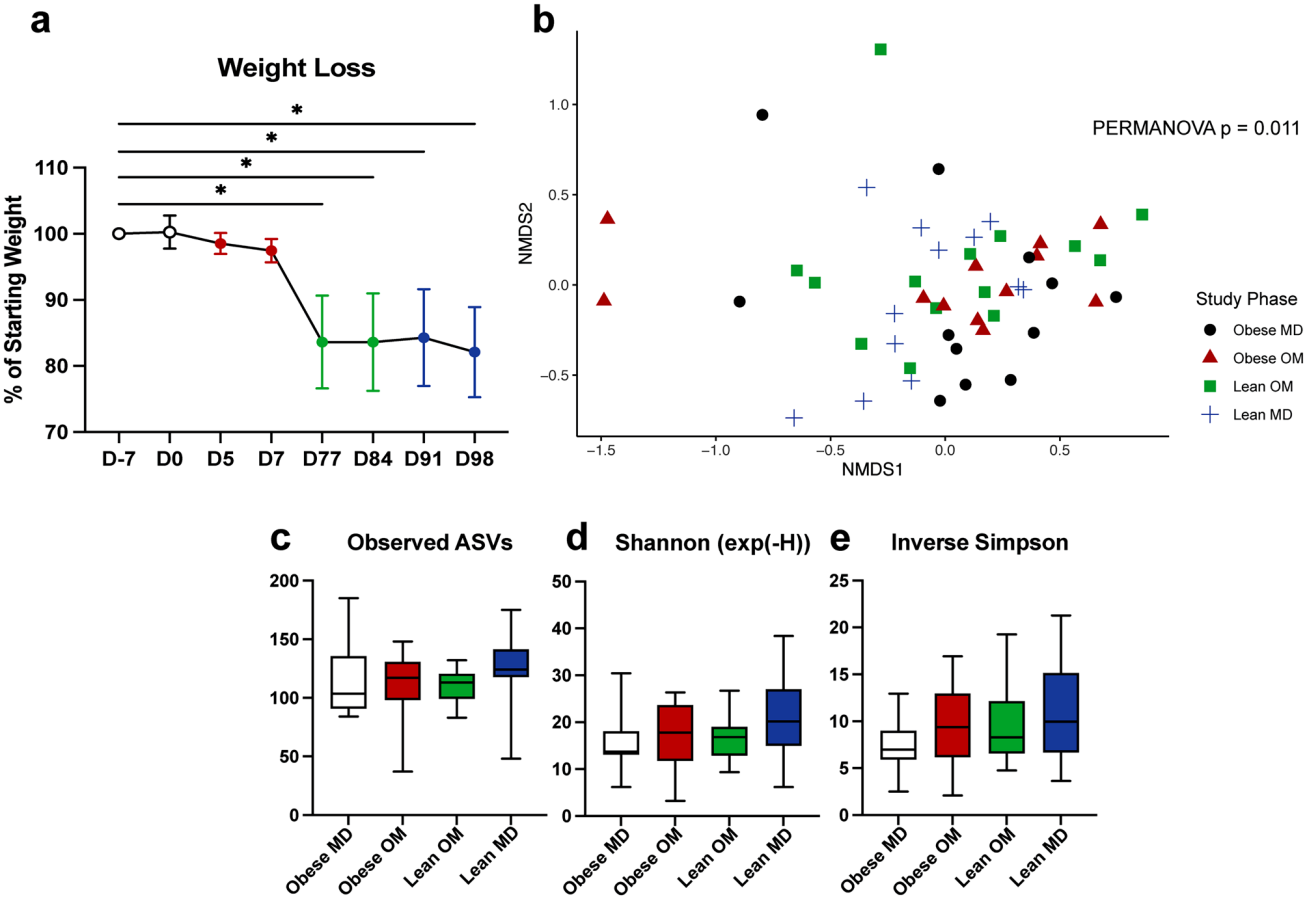
In cats, caloric restriction-induced weight loss alters gut microbiota beta diversity while alpha diversity is unchanged. (**a**) Mean weight loss as a percentage of starting weight with error bars representing standard deviation. One-way ANOVA with FDR adjusted p values (* < 0.05). (**b**) Beta diversity NMDS ordination plot using Bray–Curtis dissimilarity distances of ASVs from individual feline fecal samples. Statistical significance of differences in gut microbiota structure between study phases determined by PERMANOVA (p = 0.011). (**c**–**e**) Alpha diversity of individual feline fecal samples by study phase represented by (**c**) observed ASVs (**d**) exponential Shannon diversity index (exp(−H)) (**e**) Inverse Simpson diversity index. Box plot boxes represent interquartile range, lines within boxes represent medians, and whiskers represent range. No significant differences in alpha diversity as determined by Kruskal–Wallis test.

All cats were initially overweight or obese, with BCS measured on D-7 ranging from 7 to 9/9 (Table 1). The percentage of starting body weight was significantly reduced (FDR adj. p < 0.0001) during the Lean OM study phase by day 77 (Fig. 2a). All but one cat achieved a lean BCS (4 or 5/9) by the end of the Lean OM study phase. The one cat who did not achieve a lean BCS started with an obese BCS of 9/9 and was an overweight BCS of 7/9 by the end of the Lean OM phase. No additional significant weight change occurred during the Lean MD phase (Fig. 2a).

**Table 1:** Weight parameters during caloric restriction-induced weight loss.

Weight parameter	Obese MD (D-7)	Obese OM (D5, D7)	Lean OM (D77, D84)	Lean MD (D91, D98)
BCS, median (range)	8 (7–9)	7 (6–9)	5 (4–7)	5 (4–7)
% Starting weight, mean (SD)	100 (0)	98.0 (1.7)	83.6 (6.9)	83.2 (6.9)
Weight (kg), mean (SD)	6.0 (1.2)	5.9 (1.1)	5.1 (1.3)	5.1 (1.2)

Central tendency of continuous variables shown as mean (*SD* standard deviation) and ordinal variables as median (range).

### Caloric restriction-induced weight loss changes the gut microbial composition

The microbial community structure detected in fecal samples significantly differed by beta diversity assessed with Bray–Curtis distances between the four study phases (Fig. 2b, PERMANOVA p = 0.011). No significant differences in alpha diversity were identified between study phases using three metrics: observed ASVs, exponential Shannon diversity index, and Inverse Simpson diversity index (Fig. 2c–e).

**Figure 3:**
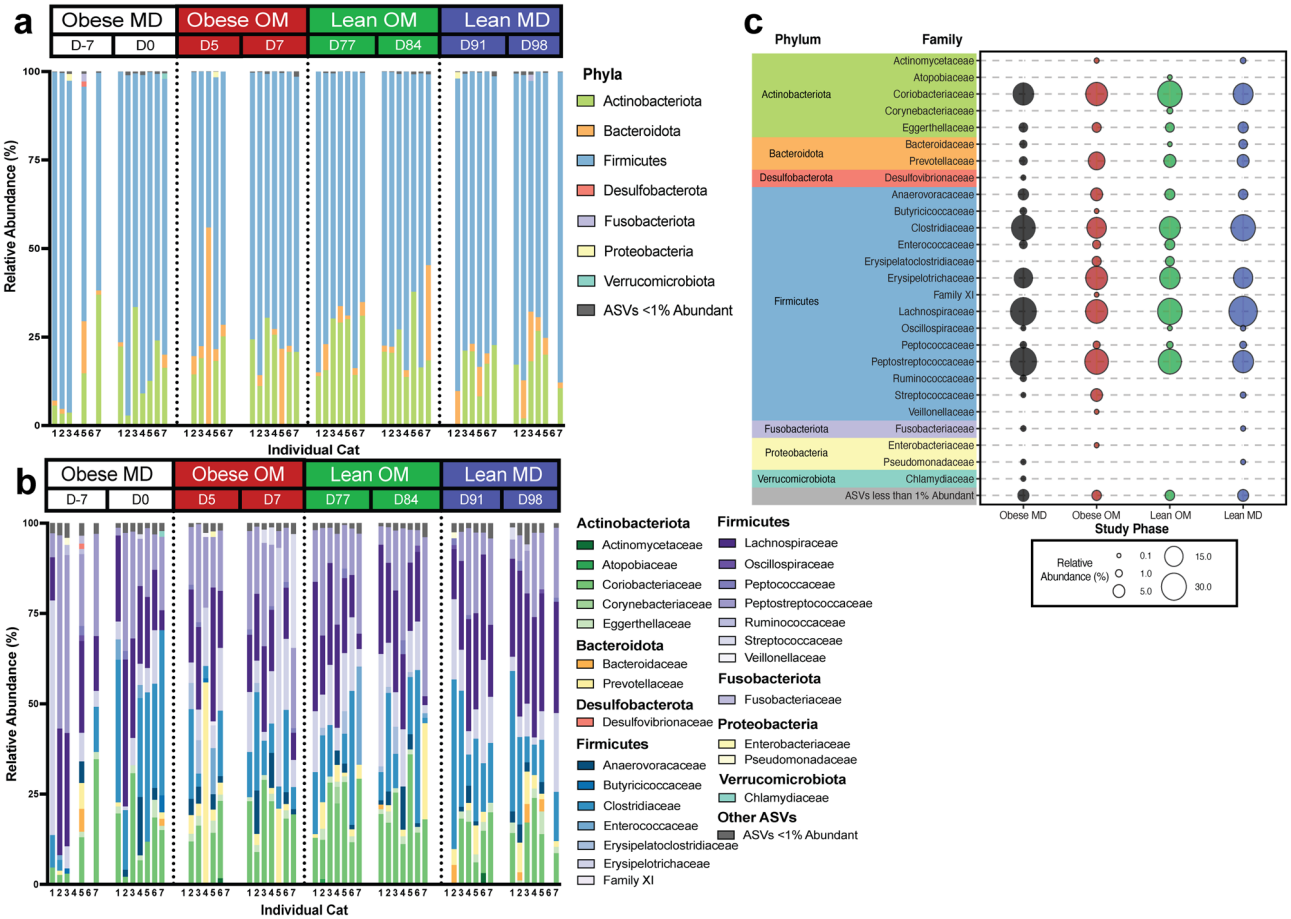
In cats, caloric restriction-induced weight loss occurs with gut microbiota changes at the phylum and family level. (**a**) Relative abundance of phyla identified in individual feline fecal samples. (**b**) Relative abundance of families identified in individual feline fecal samples. For both stacked bar plots, missing bars represent days where a fecal sample was not collected from an individual cat. (**c**) Mean relative abundance bubble plot showing the mean relative abundance of each listed family with a proportional bubble size by phase. If a family mean relative abundance was less than 0.1% abundant, there is no corresponding bubble for that study phase.

The relative abundances of ASVs detected in the fecal samples from all cats were assessed at both the phylum and family level (Fig. 3). ASVs belonging to the Firmicutes phylum were predominant in most fecal samples across all phases, though Actinobacteriota and Bacteroidota phyla were also major contributors to community composition (Fig. 3a). This agrees with previous reports of phylum-level bacterial diversity by 16S rRNA gene sequencing in healthy cats, though often members within the phylum Proteobacteria are similarly abundant to Actinobacteriota and Bacteroidota^10^. Bacteroidota was the predominant phylum represented in a fecal sample from cat 4 on study day 5, which was the first sample collected after all cats were transitioned to the calorie restricted diet. While detected in lower relative abundance, ASVs belonging to the phyla Desulfobacterota, Fusobacteriota, Proteobacteria, and Verrucomicrobiota were detected in amounts surpassing > 1% of community structure from at least one sample across the study.

ASVs belonging to 26 families were identified with a relative abundance > 1% in at least one sample. Of these families, five were within Actinobacteriota, two within Bacteroidota, one within Desulfobacterota, 14 within Firmicutes, one within Fusobacteriota, two within Proteobacteria, and one within Verrucomicrobiota (Fig. 3b,c). The relative abundances of ASVs at the family level show that a variety of families contribute to the microbial community structure and vary with both study time course and within individuals. Yet, it is difficult to single one or a few families out as consistently defining that structure.

### Known short-chain fatty acid producing bacteria, including five *Blautia* genus members, are enriched during and after caloric restriction-induced weight loss in cats

**Figure 4:**
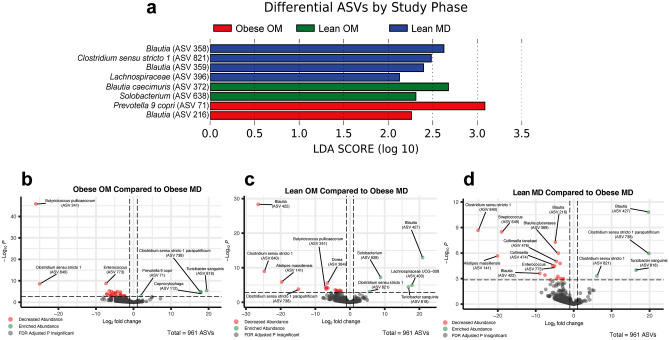
Differentially abundant ASVs are identified during and following caloric restriction-induced weight loss in cats. (**a**) Differentially abundant ASVs identified with LEfSe ordered by the study phase the ASV was enriched during and the corresponding LDA score. (**b**–**d**) Volcano plots for differentially abundant ASVs compared to Obese MD during the (**b**) Obese OM (**c**) Lean OM, and (**d**) Lean MD phases. Log_2_ fold change is plotted along the x-axis with p-values plotted along the y-axis. Vertical dashed lines represent 1 and − 1 log_2_ fold change and the dashed horizontal line represents the p-value threshold of significance (p = 0.05). Points colored in green have a positive log_2_ fold change with an FDR adjusted p value < 0.05 and points colored in red have a negative log_2_ fold change with an FDR adjusted p value < 0.05. All three volcano plots incorporate the 961 observed ASVs.

LEfSe identified differentially abundant features in any one study phase compared to all other phases (Fig. 4a). Eight ASVs were differentially abundant (LDA score > 2.0). No ASVs were differentially abundant during the Obese MD study phase. During the Obese OM study phase, *Prevotella 9 copri* (ASV 71) and a *Blautia* (ASV 216) had significant increases in relative abundance (Supplementary Fig. S3). Specifically, for *Prevotella 9 copri*, the elevation that occurred during Obese OM phase compared to Obese MD (FDR adj. p = 0.0009) remained significantly increased compared to Obese MD for the additional Lean OM (FDR adj. p = 0.0038) and Lean MD (FDR adj. p = 0.0149) phases.

During the Lean OM phase, *Blautia caecimuris* (ASV 372) (FDR adj. p = 0.0058) and *Solobacterium* (ASV 638) (FDR adj. p = 0.0006) had significant increases in relative abundance from Obese MD (Supplementary Fig. S3). The relative abundance was also increased from the Obese OM phase for *Blautia caecimuris* (ASV 372) (FDR adj. p = 0.0334) and *Solobacterium* (ASV 638) (FDR adj. p = 0.0479) during the Lean OM phase. *Blautia caecimuris* (ASV 372) then significantly decreased (FDR adj. p = 0.0117) when the maintenance diet was reintroduced during the Lean MD phase.

During the Lean MD phase, *Clostridium sensu stricto 1* (ASV 821), two *Blautia* ASVs (ASV 358 and ASV 359), and a *Lachnospiraceae* ASV (ASV 396) had significant increases in relative abundance (Supplementary Fig. S3). One of the *Blautia* genus ASVs (ASV 359) (FDR adj. p = 0.0061) and *Clostridium sensu stricto 1* (ASV 821) (FDR adj. p = 0.0079) were significantly increased from the Obese MD phase.

Log_2_ fold changes (log_2_FC) of abundance compared to Obese MD were also performed (Fig. 4b–d). Like the LEfSe analysis, *Prevotella 9 copri* (ASV 71) was significantly enriched during the Obese OM phase (log_2_FC = 2.01, FDR adj. p = 0.0254). Three additional ASVs were also enriched during the Obese OM phase: *Turicibacter sanguinis* (ASV 818) (log_2_FC = 19.5, FDR adj. p = 0.0006), *Clostridium sensu stricto 1 paraputrificum* (ASV 738) (log_2_FC = 17.8, FDR adj. p = 0.0009), and *Capnocytophaga* (ASV 112) (log_2_FC = 18.0, FDR adj. p = 0.0012). The three ASVs with the greatest log_2_FC decrease during the Obese OM phase were *Butyricicoccus pullicaecorum* (ASV 241) (log_2_FC = − 26.1, FDR adj. p < 0.0001), *Clostridium sensu stricto 1* (ASV 840) (log_2_FC = − 25.0, FDR adj. p < 0.0001), and *Enterococcus* (ASV 773) (log_2_FC = − 7.3, FDR adj. p < 0.0001).

When the Lean OM phase was compared to Obese MD, the same *Solobacterium* (ASV 638) identified in the LEfSe analysis was significantly enriched (log_2_FC = 8.8, FDR adj. p < 0.0001). The three ASVs with the greatest log_2_FC increase during the Lean OM phase compared to Obese MD were *Blautia* (ASV 427) (log_2_FC = 20.9, FDR adj. p < 0.0001), *Lachnospiraceae UCG-008* (ASV 400) (log_2_FC = 17.9, FDR adj. p = 0.0010), and *Turicibacter sanguinis* (ASV 818) (log_2_FC = 16.8, FDR adj. p = 0.0025). The three ASVs with the greatest log_2_FC decrease during the Lean OM phase were *Blautia* (ASV 422) (log_2_FC = − 26.5, FDR adj. p < 0.0001), *Clostridium sensu stricto 1* (ASV 840) (log_2_FC = − 24.7, FDR adj. p < 0.0001), and *Alistipes massiliensis* (ASV 141) (log_2_FC = − 19.7, FDR adj. p = 0.0002).

Comparing the Lean MD phase to Obese MD, the same *Clostridium sensu stricto 1* (ASV 821) identified in the LEfSe analysis was significantly enriched (log_2_FC = 6.1, FDR adj. p = 0.0201). The three ASVs with the largest log_2_FC increase were *Clostridium sensu stricto 1 paraputrificum* (ASV 738) (log_2_FC = 19.9, FDR adj. p < 0.0001), *Blautia* (ASV 427) (log_2_FC = 19.7, FDR adj. p = 0.0001), and *Turicibacter sanguinis* (ASV 818) (log_2_FC = 16.4, FDR adj. p = 0.0051). The three ASVs with the greatest log_2_FC decrease during the Lean MD phase were *Clostridium sensu stricto 1* (ASV 840) (log_2_FC = − 25.2, FDR adj. p < 0.0001), *Alistipes massiliensis* (ASV 141) (log_2_FC = − 20.1, FDR adj. p = 0.0002), and *Streptococcus* (ASV 649) (log_2_FC = − 19.0, FDR adj. p < 0.0001).

### Propionic acid production is promoted during caloric restriction-induced weight loss in cats and correlates with abundance of two bacterial species

**Figure 5:**
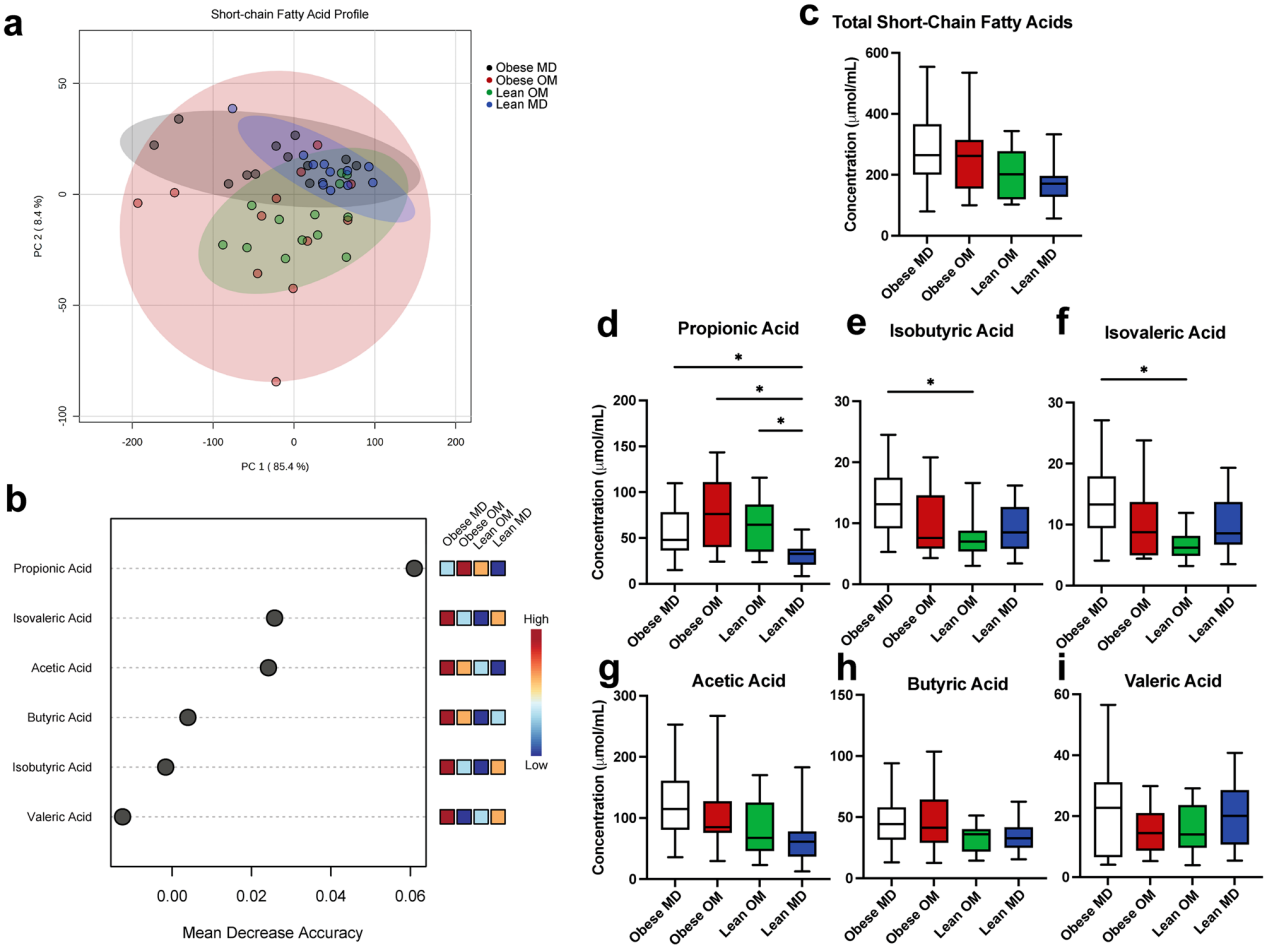
Caloric restriction-induced weight loss alters the concentrations of microbial-derived short-chain fatty acids recovered from feline feces. (**a**) PCA of individual feline fecal sample SCFA concentration (μmol/mL). Shaded ovals represent 95% confidence interval. (**b**) Significant features determined through Random Forest Classification. Points represent mean decrease in classification accuracy when permuted and colored boxes represent fecal short-chain fatty acid concentration in the indicated study phase, darker blue color indicating low concentrations and darker red color indicating high concentrations. (**c**) Box plot of the total concentration of SCFA recovered in feline feces by study phase. (**d**–**i**) Box plots of individual fecal SCFA concentrations recovered from feline feces by study phase for (**d**) propionic acid, (**e**) isobutyric acid, (**f**) isovaleric acid, (**g**) acetic acid, (**h**) butyric acid, and (**i**) valeric acid. Box plot boxes represent interquartile range, lines within boxes represent medians, and whiskers represent range. Kruskal–Wallis with FDR adjusted p values (* < 0.05).

The concentrations of SCFAs detected in individual feline fecal samples were assessed. Acetic acid concentration predominated in most instances, followed by propionic acid and butyric acid in similar concentrations, and then valeric acid, isovaleric acid, and isobutyric acid being in lower concentrations (Supplementary Figs. S4 and S5). When grouped by study phase, concentrations of fecal SCFAs display overlapping clustering by study phase without distinct separation by each study phase using principal component analysis (Fig. 5a). Propionic acid had the highest mean decrease in accuracy when permuted in a Random Forest machine learning algorithm (Fig. 5b). This demonstrated the importance of propionic acid concentration as a discriminating feature when attempting to predict which study phase a fecal sample belonged to based solely upon the detected SCFA concentrations. No difference in total SCFA concentration was identified between study phases (Fig. 5c). When individual SCFA concentrations were assessed between study phases (Fig. 5d–i), the median concentration of propionic acid was increased during the Obese OM study phase (median 76.25 μmol/mL) compared to Obese MD (median 47.9 μmol/mL) but did not reach statistical significance (FDR adj. p = 0.2191). During the Lean MD phase, the concentration of propionic acid was significantly reduced compared to Obese MD (FDR adj. p = 0.0404), Obese OM (FDR adj. p = 0.0024), and Lean OM (FDR adj. p = 0.0098) phases.

Separately from propionic acid, concentrations of isobutyric acid (FDR adj. p = 0.0324) and isovaleric acid (FDR adj. p = 0.0111) were significantly reduced from Obese MD after caloric restriction-induced weight loss had occurred in the Lean OM study phase (Fig. 5e,f). Isobutyric acid and isovaleric acid represent the two branched-chain fatty acids (BCFAs) that were measured. No statistically significant changes in concentration occurred between study phases for acetic acid, butyric acid, or valeric acid (Fig. 5g–i).

**Figure 6:**
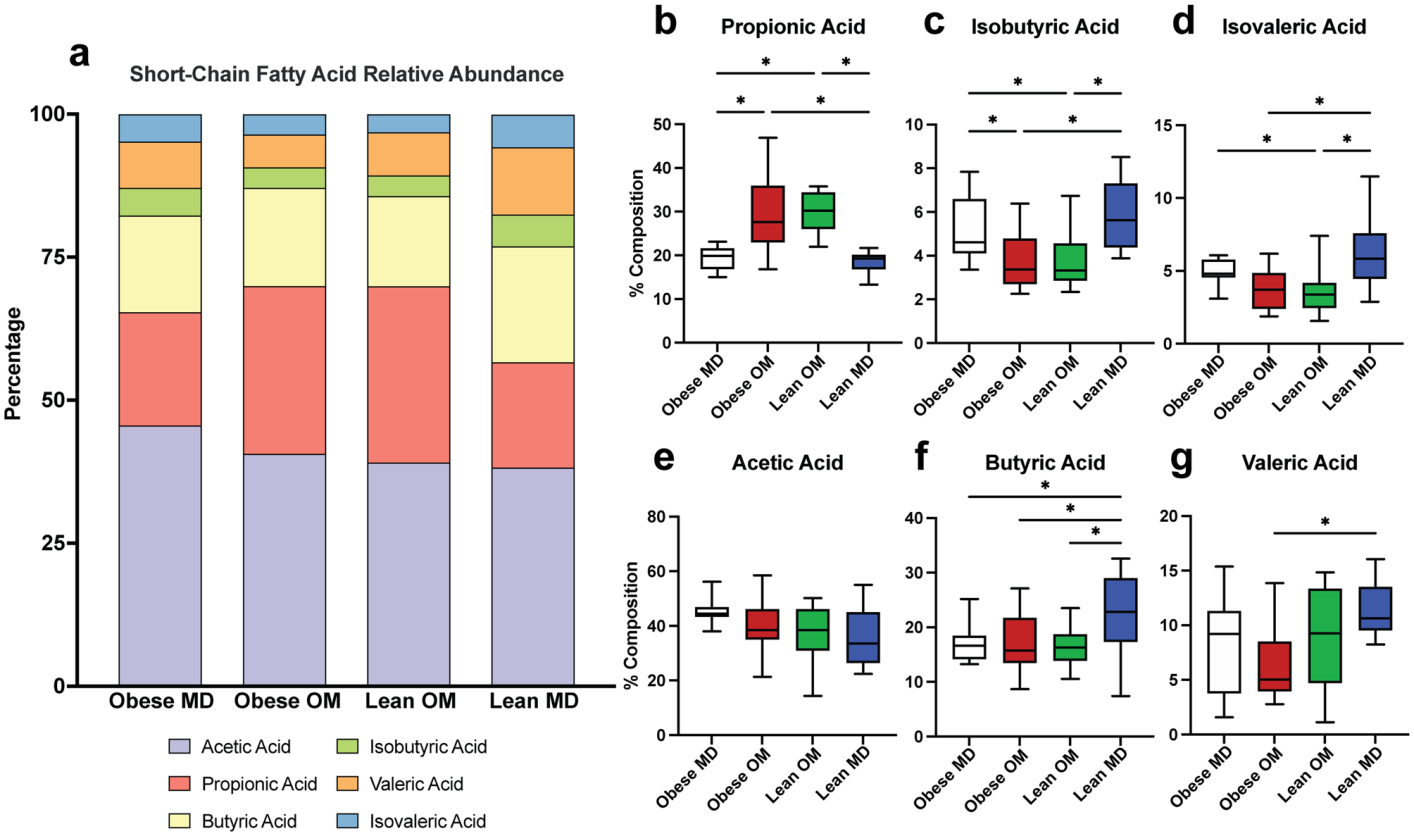
Caloric restriction-induced weight loss alters the relative composition of microbial-derived short-chain fatty acids recovered from feline feces. (**a**) Stacked bar plot of the mean composition of each SCFA concentration measured as a percentage of the total measured concentration. (**b**–**g**) Box plots of individual SCFA concentrations recovered from feline feces by study phase for (**b**) propionic acid, (**c**) isobutyric acid, (**d**) isovaleric acid, (**e**) acetic acid, (**f**) butyric acid, and (**g**) valeric acid. Box plot boxes represent interquartile range, lines within boxes represent medians, and whiskers represent range. One-way ANOVA with FDR adjusted p values (* < 0.05).

The relative composition that each of the six measured SCFAs contributed to the total concentration of SCFAs within each sample was also explored (Supplementary Fig. S6). Propionic acid contributed a significantly greater percentage of the total composition of SCFAs within feces during caloric restriction-induced weight loss in both the Obese OM (FDR adj. p < 0.0001) and Lean OM (FDR adj. p < 0.0001) phases (Fig. 6a,b). During the Lean MD phase, the composition of propionic acid was reduced to a degree not significantly different from the Obese MD study phase (FDR adj. p = 0.2535). Compositional changes also occurred with a reduction in the BCFA isobutyric acid during the Obese OM (FDR adj. p = 0.0068) and Lean OM (FDR adj. p = 0.0068) phases (Fig. 6c). Significantly reduced composition occurred for the other measured BCFA, isovaleric acid, during Lean OM phase (FDR adj. p = 0.0464) (Fig. 6d). These reductions were then followed by a significant increase of both isobutyric acid (FDR adj. p = 0.0011) and isovaleric acid (FDR adj. p = 0.0010) composition in the Lean MD phase. Though no differences in butyric acid concentration were identified between study phases (Fig. 5h), compositionally butyric acid was significantly increased in the Lean MD phase compared to Obese MD (FDR adj. p = 0.0426), Obese OM (FDR adj. p = 0.0481), and Lean OM (FDR adj. p = 0.0295) (Fig. 6f). No differences in acetic acid composition were identified between phases (Fig. 6e).

**Figure 7:**
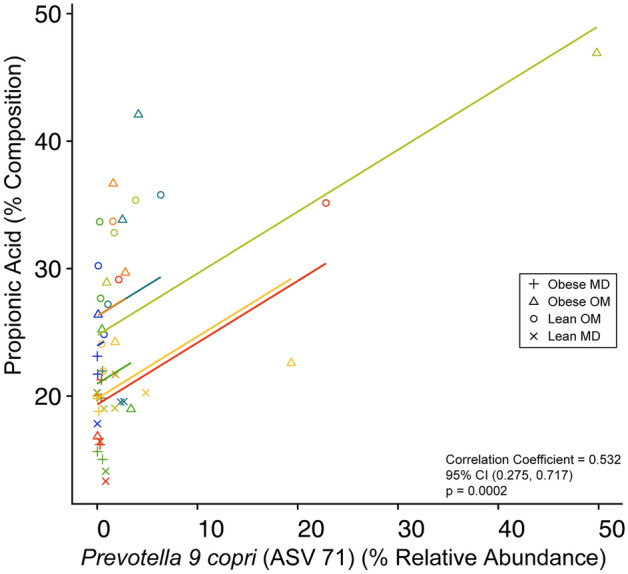
Repeated measures correlation between fecal propionic acid composition and the relative abundance of *Prevotella 9 copri* (ASV 71). Increased fecal propionic acid composition is significantly correlated with the relative abundance of differentially abundant *Prevotella 9 copri* (ASV 71). Individual cats are separated by color of the points corresponding to individual samples. Point shapes represent the corresponding study phase for the individual sample as shown in the legend on the figure (Obese MD = “+”; Obese OM = “Δ”; Lean OM = “o”; Lean MD = “x”). Lines corresponding to each individual cat are also shown by color matching to individual sample points.

Given that propionic acid was the microbial metabolite identified as enriched during caloric restriction-induced weight loss in cats, it was investigated whether changes in microbial relative abundance were correlated with the observed increase in fecal propionic acid. To answer this question in the context of caloric restriction-induced weight loss, the ASVs determined to be differentially increased in abundance during the Lean OM phase were investigated for correlation with the fecal composition of propionic acid. Fecal samples from all phases were included in the analysis, including samples with zero percent relative abundance of the ASVs. The relative abundance of *Prevotella 9 copri* (ASV 71) was significantly positively correlated with fecal propionic acid compostiion (repeated measures correlation coefficient = 0.532, 95% CI (0.275, 0.717), p = 0.0002) (Fig. 7). The relative abundance of *Blautia caecimuris* (ASV 372) was positively correlated with fecal propionic acid composition without reaching statistical significance (repeated measures correlation coefficient = 0.235, 95% CI (− 0.0703, 0.500), p = 0.129) (Supplementary Fig. S7).

## Discussion

Here, through targeted metabolomics, propionic acid was identified as a microbial-derived metabolite enriched in feces during caloric restriction-induced weight loss in cats. Microbial-derived metabolites are messengers that allow communication between the gut microbiota and host. Microbial-derived metabolites, like SCFAs, signal directly to enteroendocrine cells that in turn secrete hormones with consequential systemic metabolic effects such as gastric inhibitory polypeptide (GIP) and GLP-1^42^. Microbial-derived metabolites are also absorbed from the gastrointestinal tract and circulated through the bloodstream. At sites distant from the gastrointestinal tract, host cells sense and respond to these metabolic signals. The profile of microbial-derived metabolites the gut microbiome produces is a result of the functional capacity of the microbial community structure. A major factor that influences the gut microbial community structure in both humans and animals is diet^10,43^.

Propionic acid is sensed by free fatty acid (FFA) receptors 2 and 3, which also sense acetic acid and butyric acid^44,45^. FFA2 and FFA3 are cell surface G protein-coupled receptors that are expressed in enteroendocrine cells, bone marrow, immune cells, adipose, and pancreatic tissue^3,45–47^. Rodent models have demonstrated propionic acid is a regulator of appetite via signaling to L cells to release GLP-1 and Peptide YY (PYY)^3,47^ and promotes resistance to weight gain^48,49^ through both FFA2 and FFA3 mechanisms. Though FFA2 and FFA3 are mechanistically unstudied in cats to the authors’ knowledge, a GLP-1 analogue has been shown to promote weight loss in diabetic cats compared to placebo^50^, and FFA2 and FFA3 targeted therapeutics are of growing interest in type 2 diabetes in people given their roles in regulating insulin sensitivity and expression in pancreatic β cells^5,51^. Moreover, it is important to recognize that propionic acid itself is a substrate for gluconeogenesis in cats, providing a direct linkage to energy homeostasis^52^.

In the present study, a reduction in the BCFAs isobutyric acid and isovaleric acid occurred during caloric restriction-induced weight loss in cats. These two BCFAs are associated with obesity and insulin resistance in a murine model, directly promote hepatic glucose production, and activate mTORC1/S6K1 signaling in vitro^53^. Host mechanisms in cats that regulate glucose production and insulin sensitivity in response to BCFAs are unexplored. Increased isovaleric acid in feces from cats with CKD is reported^16^, a population of cats often characterized by a complex catabolic disease state resulting in muscle wasting and cachexia. It is unknown whether isovaleric acid metabolically contributes to CKD progression in cats, is elevated as a consequence of the disease, or is even a consequence of dietary approaches to managing feline CKD. Interestingly, isovaleric acid recovered from the urine of a cohort of human patients with type 2 diabetes was one of seven discriminating metabolites for those with diabetic kidney disease, suggesting a possible microbially-mediated metabolic link in both complex diseases^54^. Further investigation in cats may help provide additional connections between the impact of isovaleric acid concentrations on host metabolism and progression of kidney disease.

While microbial-derived metabolites are key signaling molecules for communication between gut microbes and the host^1^, this communication is highly dependent on the microbial community structure, its metabolic capabilities, and ultimate function. Though this study did not employ metagenomic sequencing capable of identifying microbial genes responsible for functional capacity, the 16S rRNA gene amplicon sequencing performed revealed how the gut microbial community structure changed during caloric restriction-induced weight loss in this cohort of cats. In several instances, separate ASVs within the same genus were identified as both significantly enriched and significantly reduced in abundance within the same study phase. This occurred with *Clostridium* *sensu stricto* 1 in the Obese OM phase and *Blautia* in the Lean OM phase when both phases were compared to the Obese MD phase. This is important to consider that with changing microbial community dynamics, even members of the same genus may have variable fitness advantages that allow them to thrive in one environment, ultimately shaping the fuctional potential of the microbiome. It is important to note that 16S rRNA gene amplicon analysis cannot be used to determine the differences in functional capacity between identified differentially abundant members of the same genus, to do this metagenomics would be required. When the metabolomic outcome of increased propionic acid was investigated, the two microbes identified to correlate with the increased composition of propionic acid were *Prevotella 9 copri* and *Blautia caecimuris*, though the correlation with *Blautia caecimuris* did not reach statsitical signficance.

The genus *Prevotella* is often most abundant within healthy feline fecal samples and was recently proposed as one of 30 genera constituting the core feline gut microbiome^55^. Previous work has also correlated *Prevotella* with propionic acid composition in feline feces when healthy cats were fed a variety of kibble and raw diets supplemented with fiber^56^. However, in the present study *Prevotella 9 copri*, which has been reported to lack the cellular machinery in carbon metabolism to produce propionic acid itself but rather produces succinate instead^57^, was positively correlated with fecal propionic acid in obese cats undergoing caloric restriction-induced weight loss. This discrepancy highlights the need for multi-omics approaches, where metagenomics and metabolomics performed in tandem may provide clarity into underlying mechanism. In this case, defining the metagenomic assembled genome of *Prevotella 9 copri* could describe SCFA metabolism capabilities specific to feline gut microbes. Herein, it is impossible to evaluate a causative mechanism behind the positive correlation of *Prevotella 9 copri* and propionic acid. Speculatively, *Prevotella 9 copri* could be involved in a more complicated metabolic cross-feeding with other members of the gut microbiota that yields net propionic acid production, even if the production is not directly from *Prevotella 9 copri* metabolism. *Prevotella 9 copri* may be a sentinel feature in a larger network of microbial relationships responsible for increased propionic acid. Though unlikely, strain-level variation in *Prevotella 9 copri* could provide the necessary cellular machinery for direct propionic acid production, and the existing data is largely sourced from the lab strain DSM 18205^57^.

The other microbe positively correlated with propionic acid composition was *Blautia caecimuris*. Though the positive correlation between relative abundance of *Blautia caecimuris* and fecal propionic acid composition did not reach statistical significance in the current study, it is an intriguing finding to further explore in a translational biological context. The genus *Blautia* is another proposed to be one of 30 core genera in the healthy feline gut microbiome^55^. Four additional *Blautia* ASVs were enriched in the present study either during or following caloric restriction-induced weight loss in cats. *Blautia* has garnered attention for potential application as a therapeutic probiotic in several capacities given its broad ability to perform biotransformations and generate beneficial metabolites, including SCFAs^58^. Within the realm of obesity and type 2 diabetes, *Blautia wexlerae* was identified in a cross-sectional study of Japanese adults to be inversely related to obesity and type 2 diabetes^59^. To evaluate this relationship further, *Blautia wexlerae* was administered in a murine obesity model, resulting in higher fecal propionic acid and amelioration of obesity^59^. Interestingly, *Blautia wexlerae* lacked the machinery to directly produce propionic acid itself^59^. This is intriguing given that in the present study, *Blautia caecimuris* was positively correlated with fecal propionic acid concentration and its closest phylogenetic neighbor is *B. wexlerae*^60^. Though impossible to generate a causative link from our data between *Blautia caecimuris* and increased fecal propionic acid, there appears to be a cross-species conserved association between these two features in the context of weight loss and obesity. This link provides additional support for obese cats acting as a translational model for human obesity.

Diet is the major nutrient source for the gut microbiota and often the driver of microbial community alterations^10,43^. Here, the veterinary therapeutic weight loss diet fed to all obese cats was high in protein and fiber concentrations (Supplementary Table S1). Feline diets with similar nutrient profiles exist commercially. Total dietary fiber (TDF) is comprised of both soluble and insoluble fibers, impacting solubility and fermentability, and is the best measure for comparing dietary fiber content between diets^61,62^. However, TDF was not available for the maintenance diet used in this study, so crude fiber was listed for comparison. Crude fiber does not account for soluble fibers that are often fermentable and contribute to SCFA production^61,62^. Both the type and amount of fiber within the diet impacts potential gut microbial metabolism and subsequent SCFA generation. Similarly, amino acid composition of protein dictates the ultimate breakdown products of protein metabolism by the gut microbiota. Proteins that contain more branched-chain amino acids will promote BCFA production, since microbial metabolism of branched-chain amino acids creates BCFAs. Fecal SCFAs in this study should be viewed in light of the fed diet composition and cannot be generalized to all cats who lose weight when receiving another reduced calorie diet lacking a similar nutrient profile. Additionally, all weight loss in this study occurred through intentional calorie restriction overseen by a board-certified veterinary internist and nutritionist, and use of other caloric restriction-induced weight loss approaches should always be employed with veterinary oversight.

Host factors also influence gut microbial community structure, including systemic inflammation and local gastrointestinal mucosal immune responses which directly interface with the gut microbiota^63,64^. Broadly, obesity can be classified as a pro-inflammatory state characterized by cytokine responses contributing to insulin resistance^65,66^. Interventions in people including diet, exercise, and weight loss can reverse the pro-inflammatory state and improve insulin sensitivity^67^. Similarly, reversing obesity through modulation of dietary intake has been shown to improve insulin sensitivity in cats^68^, and adipose tissue in obese cats has been shown to have higher pro-inflammatory cytokine expression of TNFα than lean cats^69^. This highlights the cross-species similarity between cats and humans in this context. Measures of systemic or local inflammation were not performed in the present study, but changes to host inflammation in the cats may have contributed to changes in gut microbial community structure and function. Collectively, factors beyond diet alone are also likely contributing to alterations in this complex ecosystem. In the present study, the significant reduction in propionic acid concentration during the Lean MD phase compared Obese MD is notable since cats were eating the same maintenance diet during both phases, suggesting that diet alone was not the only factor contributing to fecal propionic acid concentration.

Persistent changes to microbial community members when the maintenance diet was reintroduced in the Lean MD phase of this study provides evidence for factors beyond diet alone impacting microbial community structure. Changes to the microbial community from Obese MD that were first identified in the Lean OM phase and persisted in the Lean MD phase were increased log_2_FC of *Blautia* (ASV 427) and *Turicibacter* sanguinis (ASV 818) and decreased log_2_FC of a *Clostridium sensu stricto 1* (ASV 840) and *Alistipes massiliensis* (ASV 141). *Turicibacter sanguinis* has recently been described to be enriched via a host-secreted serotonin mechanism^70^, and propionic acid can stimulate host serotonin release^71,72^. While serotonin was not investigated in the present study, *Turicibacter sanguinis* could have gained a fitness advantage from propionic acid stimulated serotonin release allowing niche establishment within this ecosystem. *Alistipes massiliensis* has been shown to be enriched in cecal content of mice fed a high-fat diet producing an insulin resistant and pro-inflammatory state, including elevated IL-6 cytokine expression^73^. Though a relatively newer genus with 13 known species, *Alistipes* more generally has been associated with altered human host metabolism as one known species, *A. ihumii*, was originally recovered from feces of a patient with anorexia nervosa and a separate species, *A. obesi*, isolated from an obese person^74^. The present study finds that caloric restriction-induced weight loss of obese cats results in a significant decrease of *Alistipes massiliensis* both when a calorie-reduced weight loss diet is fed and for at least 2 weeks after returning to a maintenance diet.

The present study was limited in scope by the number of animals (n = 7), acquiring only two fecal samples per cat per study phase, and the post-hoc analysis of the gut microbiome from the original study^20^. Study design would have differed if specifically tailored to assess the microbiome. Still, there is clarity from the post-hoc analysis performed when the data are viewed across the four phases of study, and individual variation does not preclude assessing the microbiota and metabolomic data presented. When attempting to integrate a third layer of multi-omic data where the same cats were shown to have improved glucose-dependent insulinotropic peptide responses following caloric restriction-induced weight loss^20^, there was not adequate statistical power to draw meaningful conclusions from this post-hoc analysis. Striving for robust study design that allows for such layered multi-omic approaches to address mechanistic questions in complex microbiome interactions will provide new insights into the ways microbial-derived metabolites impact states of health and disease.

In summary, caloric restriction-induced weight loss in cats alters the gut microbial community structure to a state that promotes the microbially-derived metabolite propionic acid. The change in microbial community occurred as a direct result of diet as well as likely changes in host-related mechanisms that shift during weight loss and remain to be further identified and studied. The bacterial species *Prevotella 9 copri* was found to significantly positively correlate with increased fecal propionic acid, a microbial metabolite characterized in other mammalian species within the context of obesity and type 2 diabetes^3,4,38,45,47,49,59^. This cross-species conservation of findings highlights the potential application of obese cats as a translational model to further investigate the role of the gut microbiome and microbial-derived metabolites in the context of obesity and type 2 diabetes aimed at improving feline and human health.

### Supplementary Information

Supplementary Information.

## Data Availability

Sequence data is publicly available through Sequence Read Archive and can be found using the project ID PRJNA1004671.
