# Supplementary material for: Gut microbiota promoting propionic acid production accompanies caloric restriction-induced intentional weight loss in cats
Source: Sci Rep. 2024 May 24;14:11901. doi: 10.1038/s41598-024-62243-4 (PMC11126632; doi:10.1038/s41598-024-62243-4)
Supplement: Supplementary file 1 — Supplementary Information. [file 41598_2024_62243_MOESM1_ESM.docx]

| **Diet Component** | **Friskies Classic Pate Mariner’s Catch (canned)** | **Purina OM (canned)** | **Hill’s a/d (canned)** |
| --- | --- | --- | --- |
| Kcal/kg | 1,113 | 835 | 1,151 |
| Protein (g/100 kcal) | 10.8 | 13.2 | 9.2 |
| Fat (g/100 kcal) | 6.6 | 3.8 | 6.3 |
| Carbohydrate (g/100 kcal) | 1.8 | 6.1 | 3.2 |
| Crude Fiber (g/100 kcal) | 0.1 | 3.0 | 0.3 |

**Supplementary Table S1:**

Macronutrient composition of study diets. All nutrients are presented on a g/100 kcal basis.

| Obese MD (Phase 1) | | | | | | | | | | | | |  |  |  |
| --- | --- | --- | --- | --- | --- | --- | --- | --- | --- | --- | --- | --- | --- | --- | --- |
| **Cat** | **BCS (End of Phase)** | **Avg Weight (During Phase)** | **Avg Kcal Ingest per Day** | **Cans Ingest per Day** | **Lean BW** | | **Lean BW RER** | | | **RER Multiplier Factor** | | |  |  |  |
|  |  |  |  |  |  | |  | | |  | | |  |  |  |
| **1** | **7** | 5.2 | 269.04 | 1.52 | 4.16 | | 203.90 | | | 1.32 | | |  |  |  |
| **2** | **8** | 6.4 | 249.57 | 1.41 | 4.48 | | 215.55 | | | 1.16 | | |  |  |  |
| **3** | **7** | 5.1 | 279.66 | 1.58 | 4.08 | | 200.95 | | | 1.39 | | |  |  |  |
| **4** | **8** | 5.2 | 212.40 | 1.20 | 3.64 | | 184.47 | | | 1.15 | | |  |  |  |
| **5** | **7** | 5.4 | 192.93 | 1.09 | 4.32 | | 209.75 | | | 0.92 | | |  |  |  |
| **6** | **9** | 7.3 | 316.83 | 1.79 | 4.38 | | 211.94 | | | 1.49 | | |  |  |  |
| **7** | **8** | 6.25 | 217.71 | 1.23 | 4.38 | | 211.75 | | | 1.03 | | |  |  |  |
|  | Obese OM (Phase 2) | | | | | | | | | | | |  |  |  |
| **Cat** | **BCS (End of Phase)** | **Avg Weight (During Phase)** | **Avg Kcal Ingest per Day** | **Cans Ingest per Day** | **Lean BW** | | **Lean BW RER** | | | **RER Multiplier Factor** | | |  |  |  |
|  |  |  |  |  |  | |  | | |  | | |  |  |  |
| **1** | 7 | 5.1 | 226.98 | 1.94 | 4.16 | | 203.90 | | | 1.11 | | |  |  |  |
| **2** | 8 | 6.4 | 226.98 | 1.94 | 4.48 | | 215.55 | | | 1.05 | | |  |  |  |
| **3** | 7 | 5 | 230.49 | 1.97 | 4.08 | | 200.95 | | | 1.15 | | |  |  |  |
| **4** | 8 | 5.1 | 186.03 | 1.59 | 3.64 | | 184.47 | | | 1.01 | | |  |  |  |
| **5** | 6 | 5.3 | 176.67 | 1.51 | 4.32 | | 209.75 | | | 0.84 | | |  |  |  |
| **6** | 9 | 7.4 | 303.03 | 2.59 | 4.38 | | 211.94 | | | 1.43 | | |  |  |  |
| **7** | 7 | 6.05 | 190.71 | 1.63 | 4.375 | | 211.75 | | | 0.90 | | |  |  |  |
|  | Lean OM (Phase 3) | | | | | | | | | | | | | | |
| **Cat** | **BCS (End of Phase)** | **Avg Weight (End Phase)** | **Avg Kcal Provided Per Day** | **Multiplier** | **Average Percent Restriction** | | | **Lean BW** | | | **Lean BW RER** | | | **RER Multiplier Factor** | |
|  |  |  |  |  |  | | |  | | |  | | |  | |
| **1** | 5 | 4.3 | 192.93 | 0.85 | 15 | | | 4.16 | | | 203.90 | | | 0.95 | |
| **2** | 6 | 5 | 192.93 | 0.85 | 15 | | | 4.48 | | | 215.55 | | | 0.90 | |
| **3** | 5 | 4 | 195.92 | 0.85 | 15 | | | 4.08 | | | 200.95 | | | 0.97 | |
| **4** | 5 | 4.2 | 158.13 | 0.85 | 15 | | | 3.64 | | | 184.47 | | | 0.86 | |
| **5** | 4 | 4.5 | 150.17 | 0.85 | 15 | | | 4.32 | | | 209.75 | | | 0.72 | |
| **6** | 7 | 5.9 | 250.00 | 0.825 | 17.5 | | | 4.38 | | | 211.94 | | | 1.18 | |
| **7** | 5 | 4.6 | 171.64 | 0.9 | 10 | | | 4.375 | | | 211.75 | | | 0.81 | |
|  | Lean MD (Phase 4) | | | | | | | | | | | | | |  |
| **Cat** | **BCS (End of Phase)** | **Avg Weight (End Phase)** | **Avg Kcal Provided Per Day** | **Multiplier** | **Average Percent Restriction** | **Lean BW** | | | **Lean BW RER** | | | **RER Multiplier Factor** | | |  |
|  |  |  |  |  |  |  | | |  | | |  | | |  |
| **1** | 5 | 4.3 | 204.28 | 0.9 | 15 | 4.16 | | | 203.90 | | | 1.00 | | |  |
| **2** | 5 | 5 | 204.28 | 0.9 | 15 | 4.48 | | | 215.55 | | | 0.95 | | |  |
| **3** | 5 | 4 | 207.44 | 0.9 | 15 | 4.08 | | | 200.95 | | | 1.03 | | |  |
| **4** | 5 | 4.2 | 176.73 | 0.95 | 7.5 | 3.64 | | | 184.47 | | | 0.96 | | |  |
| **5** | 5 | 4.3 | 167.84 | 0.95 | 15 | 4.32 | | | 209.75 | | | 0.80 | | |  |
| **6** | 7 | 5.9 | 250.00 | 0.825 | 17.5 | 4.38 | | | 211.94 | | | 1.18 | | |  |
| **7** | 5 | 4.5 | 185.94 | 0.975 | 10 | 4.375 | | | 211.75 | | | 0.88 | | |  |

**Supplementary Table S2:**

BCS and body weight data for individual cats across study phases with average caloric intake and energy intake as a factor of RER reported.

**Supplementary Figure S3:** Eight differentially abundant ASVs identified with LEfSe during and following diet-induced weight loss. (a-h) Percentage relative abundance by study phase for differentially abundant ASVs (a) *Prevotella 9 copri* (ASV 71), (b) *Blautia* (ASV 216), (c) *Blautia caecimuris* (ASV 372), (d) *Solobacterium* (ASV 638), (e) *Blautia* (ASV 358), (f) *Clostridium sensu stricto 1* (ASV 821), (g) *Blautia* (ASV 359), and (h) *Lachnospiraceae* (ASV 396). Box plot boxes represent interquartile range, lines within boxes represent medians, and whiskers represent range. Kruskal-Wallis with FDR adjusted p values (* < 0.05).

**Supplementary Figure S4:**

Supervised heat map of individual cat fecal samples shaded according to concentration (μmol/mL) of the indicated fecal short chain fatty acid. Higher concentrations represented by darkening shades of red and lower concentrations by darkening shades of blue. Fecal samples are ordered left to right according to study phase with individual samples labeled by cat number followed by the study day.

**Supplementary Figure S5:**

Stacked bar plot of absolute fecal short-chain fatty acid concentrations (μmol/mL) for individual cats.

**Supplementary Figure S6:**

Stacked bar plot of relative composition of fecal short-chain fatty acid concentrations for individual cats.

**Supplementary Figure S7:**

Repeated measures correlation between fecal propionic acid composition and the relative abundance of *Blautia caecimuri*s (ASV 372). Increased fecal propionic acid composition is not statistically significantly positively correlated with the relative abundance of differentially abundance *Blautia caecimuri*s (ASV 372). Individual cats are separated by color of the points corresponding to individual samples. Point shapes represent the corresponding study phase for the individual sample as shown in the legend on the figure (Obese MD = “+”; Obese OM = “Δ”; Lean OM = “o”; Lean MD = “x”). Lines corresponding to each individual cat are also shown by color matching to individual sample points.
